# REFLECTIONS FROM THE PERSPECTIVE OF A STATE POLICY MAKER

**DOI:** 10.1093/geroni/igz038.2338

**Published:** 2019-11-08

**Authors:** LaRhae Knatterud

**Affiliations:** Minnesota Department of Human Services, St. Paul, Minnesota, United States

## Abstract

From her perspective as Director of Systems Transformation, Minnesota Department of Human Services, and her role as planner and policy analyst in state agencies, this presentation briefly comment on the implications of the findings. Using thepory of disruptive innvoation, she will suggest next steps for these results.

